# Supplementary material for: Solving the MCM paradox by visualizing the scaffold of CMG helicase at active replisomes
Source: Nat Commun. 2022 Oct 14;13:6090. doi: 10.1038/s41467-022-33887-5 (PMC9568601; doi:10.1038/s41467-022-33887-5)
Supplement: Supplementary file 1 — Supplementary Information [file 41467_2022_33887_MOESM1_ESM.pdf]

SUPPLEMENTARY INFORMATION

Solving the MCM paradox by visualizing the scaffold of CMG helicase at active replisomes

Hana Polasek-Sedlackova[1,2*], Thomas C. R. Miller[3], Jana Krejci[2], Maj-Britt Rask[1] and Jiri Lukas[1*]

[1] *Protein Signaling Program, Novo Nordisk Foundation Center for Protein Research, Faculty of Health and Medical Sciences, University of Copenhagen, Copenhagen, Denmark*

[2] *Department of Cell Biology and Epigenetics, Institute of Biophysics, Czech Academy of Sciences, Brno, Czech Republic*

[3] *Center for Chromosome Stability, Department of Cellular and Molecular Medicine, Faculty of Health and Medical Sciences, University of Copenhagen, Copenhagen, Denmark*

*Corresponding authors: H.P-S. (polasek-sedlackova@ibp.cz), J.L. (jiri.lukas@cpr.ku.dk)

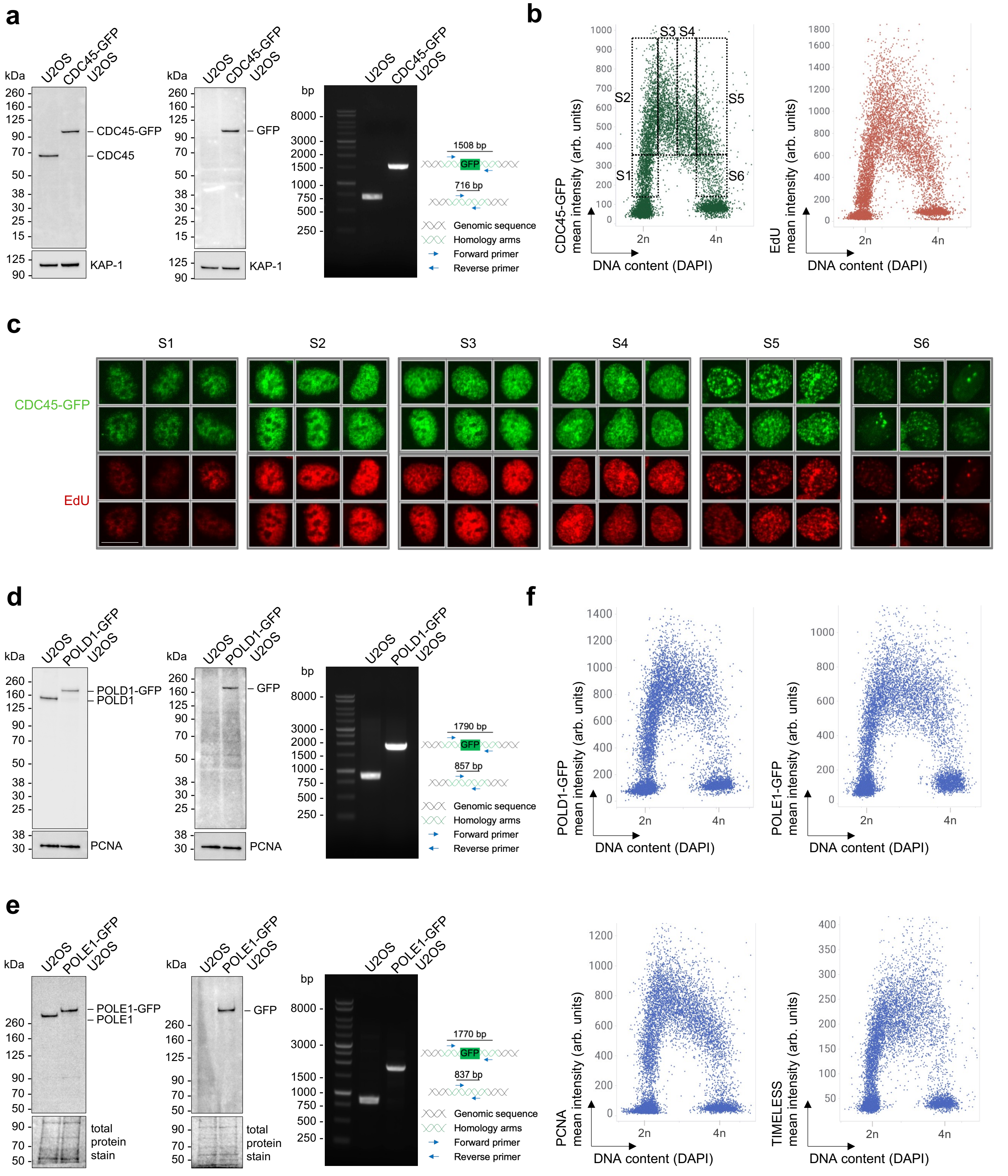

a
U2OS
CDC45-GFP
U2OS
kDa
CDC45-GFP
CDC45
GFP
KAP-1
bp
1508 bp
716 bp
GFP
Genomic sequence
Homology arms
Forward primer
Reverse primer
b
S1
S2
S3
S4
S5
S6
CDC45-GFP mean intensity (arb. units)
EdU mean intensity (arb. units)
DNA content (DAPI)
2n
4n
c
S1
S2
S3
S4
S5
S6
CDC45-GFP
EdU
d
U2OS
POLD1-GFP
U2OS
POLD1-GFP
POLD1
GFP
PCNA
1790 bp
857 bp
e
U2OS
POLE1-GFP
U2OS
POLE1-GFP
POLE1
GFP
total protein stain
1770 bp
837 bp
f
POLD1-GFP mean intensity (arb. units)
POLE1-GFP mean intensity (arb. units)
PCNA mean intensity (arb. units)
TIMELESS mean intensity (arb. units)
DNA content (DAPI)

Supplementary Fig. 1 | Generation and characterization of endogenously tagged replisome components. a, Left and middle, western blotting of U2OS cells and their derivative with endogenously tagged CDC45-GFP immunoblotted with CDC45 or GFP antibody as indicated. Right, junction PCR showing homozygous endogenous tagging of CDC45-GFP; n = 3 biological replicates. **b,** QIBC of CDC45-GFP cells pulse-labelled with EdU and immunostained for chromatin-bound GFP as indicated. Nuclear DNA was counterstained by DAPI. Dashed boxes mark the S phase stages (S1-S6) based on CDC45-GFP and DAPI intensities; n ≈10,000 cells for each condition; arb. (arbitrary) units. **c,** Representative QIBC galleries of chromatin-bound CDC45-GFP and EdU throughout the S phase sub-stratified as in (**b**). Scale bar, 20 μm. **d,** Left and middle, western blotting of U2OS cells and their derivative with endogenously tagged POLD1-GFP immunoblotted with POLD1 or GFP antibody as indicated. Right, junction PCR showing homozygous endogenous tagging of POLD1-GFP cells; n = 3 biological replicates. **e,** Left and middle, western blotting of U2OS cells and their derivative with endogenously tagged POLE1-GFP immunoblotted with POLE1 or GFP antibody as indicated. Right, junction PCR showing homozygous endogenous tagging of POLE1-GFP cells; n = 3 biological replicates. **f,** Top, QIBC of POLD1-GFP cells (left) and POLE1-GFP cells (right) immunostained for chromatin-bound GFP. Bottom, QIBC of U2OS cells immunostained for chromatin-bound PCNA (left) or TIMELESS (right). DNA was counterstained by DAPI; n ≈10,000 cells per condition; arb. (arbitrary) units. Source data are provided as a Source data file.

a

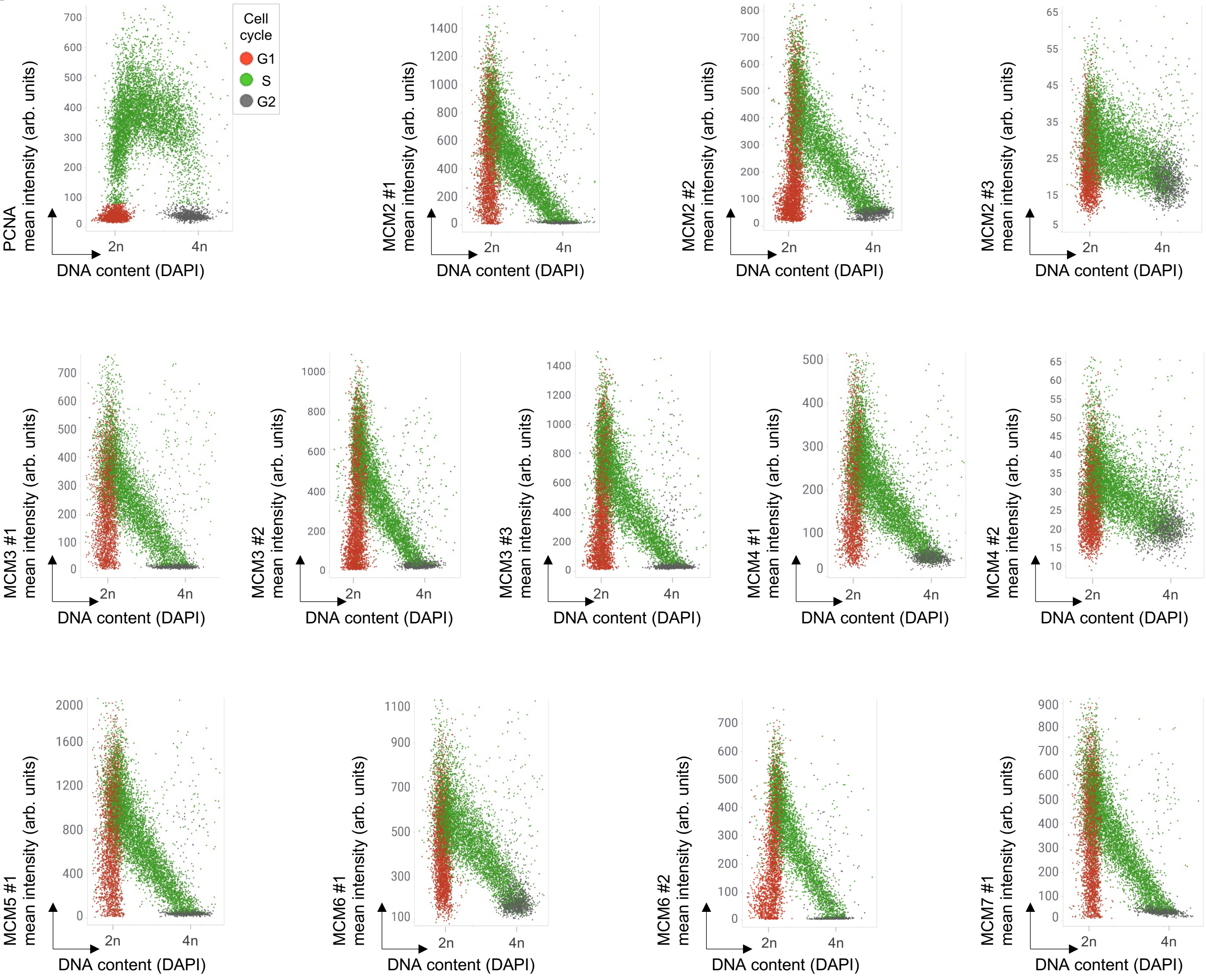

Cell cycle
G1
S
G2
PCNA mean intensity (arb. units)
MCM2 #1 mean intensity (arb. units)
MCM2 #2 mean intensity (arb. units)
MCM2 #3 mean intensity (arb. units)
MCM3 #1 mean intensity (arb. units)
MCM3 #2 mean intensity (arb. units)
MCM3 #3 mean intensity (arb. units)
MCM4 #1 mean intensity (arb. units)
MCM4 #2 mean intensity (arb. units)
MCM5 #1 mean intensity (arb. units)
MCM6 #1 mean intensity (arb. units)
MCM6 #2 mean intensity (arb. units)
MCM7 #1 mean intensity (arb. units)
DNA content (DAPI)
2n
4n

Supplementary Fig. 2 | Immunostained MCMs progressively dissociate from chromatin throughout the S phase. a, QIBC of CDC45-GFP cells immunostained for chromatin-bound PCNA and the indicated MCMs. Nuclear DNA was counterstained by DAPI. PCNA was used as an additional cell cycle marker; n ≈10,000 cells per condition (except n ≈8,000 cells for MCM3 #1, n ≈7,000 cells for MCM6 #2); arb. (arbitrary) units. Source data are provided as a Source data file.

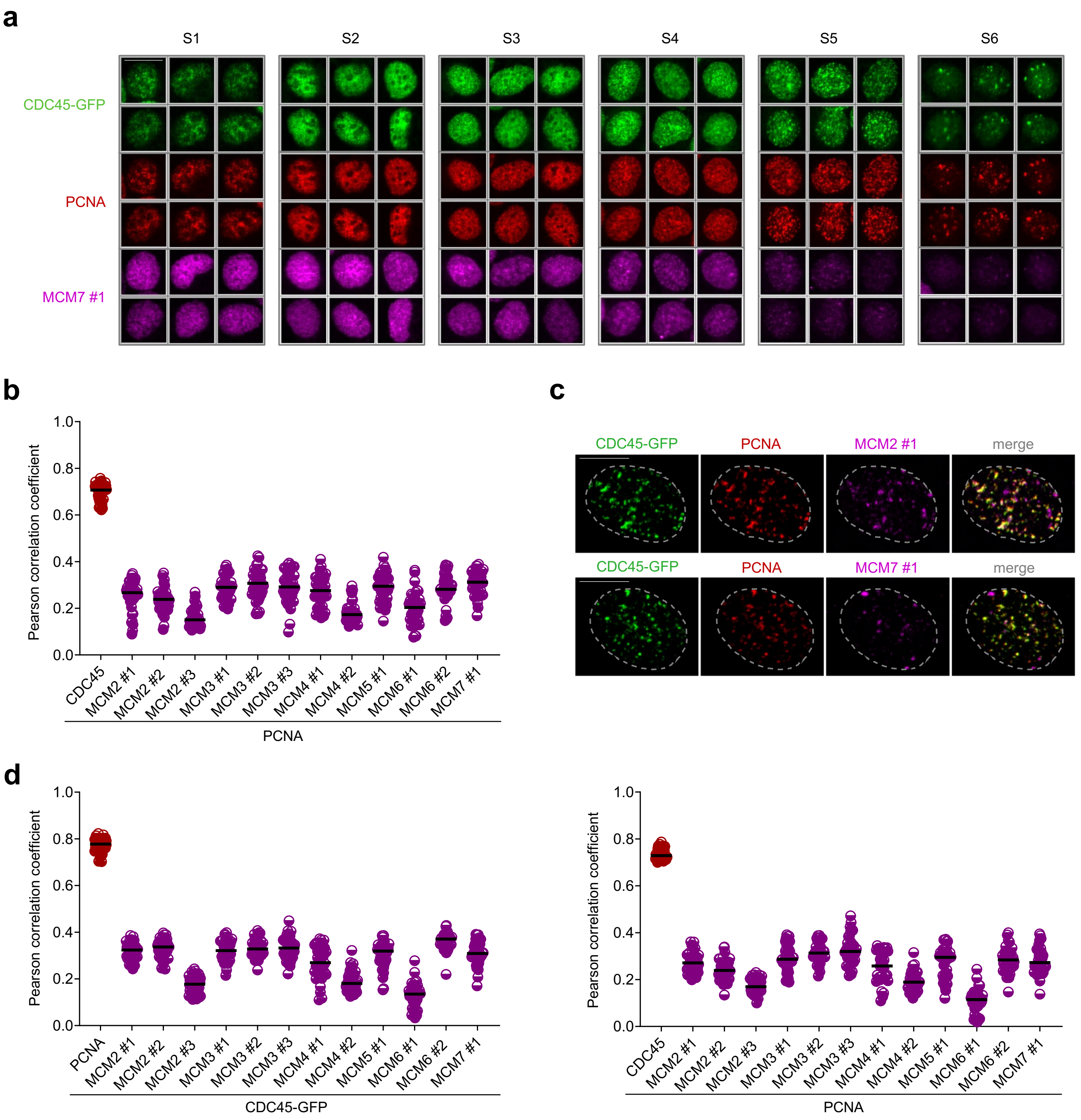
a
S1
S2
S3
S4
S5
S6
CDC45-GFP
PCNA
MCM7 #1
b
Pearson correlation coefficient
1.0
0.8
0.6
0.4
0.2
0.0
CDC45
MCM2 #1
MCM2 #2
MCM2 #3
MCM3 #1
MCM3 #2
MCM3 #3
MCM4 #1
MCM4 #2
MCM5 #1
MCM6 #1
MCM6 #2
MCM7 #1
PCNA
c
CDC45-GFP
PCNA
MCM2 #1
merge
CDC45-GFP
PCNA
MCM7 #1
merge
d
Pearson correlation coefficient
PCNA
MCM2 #1
MCM2 #2
MCM2 #3
MCM3 #1
MCM3 #2
MCM3 #3
MCM4 #1
MCM4 #2
MCM5 #1
MCM6 #1
MCM6 #2
MCM7 #1
CDC45-GFP
Pearson correlation coefficient
CDC45
MCM2 #1
MCM2 #2
MCM2 #3
MCM3 #1
MCM3 #2
MCM3 #3
MCM4 #1
MCM4 #2
MCM5 #1
MCM6 #1
MCM6 #2
MCM7 #1
PCNA

Supplementary Fig. 3 | Higher stringency of pre-extraction does not increase the ability of antibodies to detect MCMs in active replisomes. **a,** Representative QIBC galleries of chromatin-bound CDC45-GFP, PCNA and immunostained MCM7 #1 throughout the S phase sub-stratified as in (Supplementary Fig. 1b). Scale bar, 20 µm. **b,** Colocalization analysis of immunostained MCMs with RFs visualized by immunostained PCNA in S5 stage of S phase. Horizontal lines are medians; n = 40 cells per condition. **c,** Representative MIP confocal images of chromatin-bound CDC45-GFP, PCNA and MCM2 or MCM7 detected by indicated antibodies in the S5 stage of S phase sub-stratified as in (Supplementary Fig. 1b). Cells were pre-extracted before fixation with CSK buffer. Scale bar, 10 µm. **d,** Left and right, colocalization analysis of immunostained MCMs with RFs visualized by endogenously tagged CDC45-GFP (left panel) or immunostained PCNA (right panel) in the S5 stage of S phase. Horizontal lines are medians; n = 40 cells per condition. Source data are provided as a Source data file.

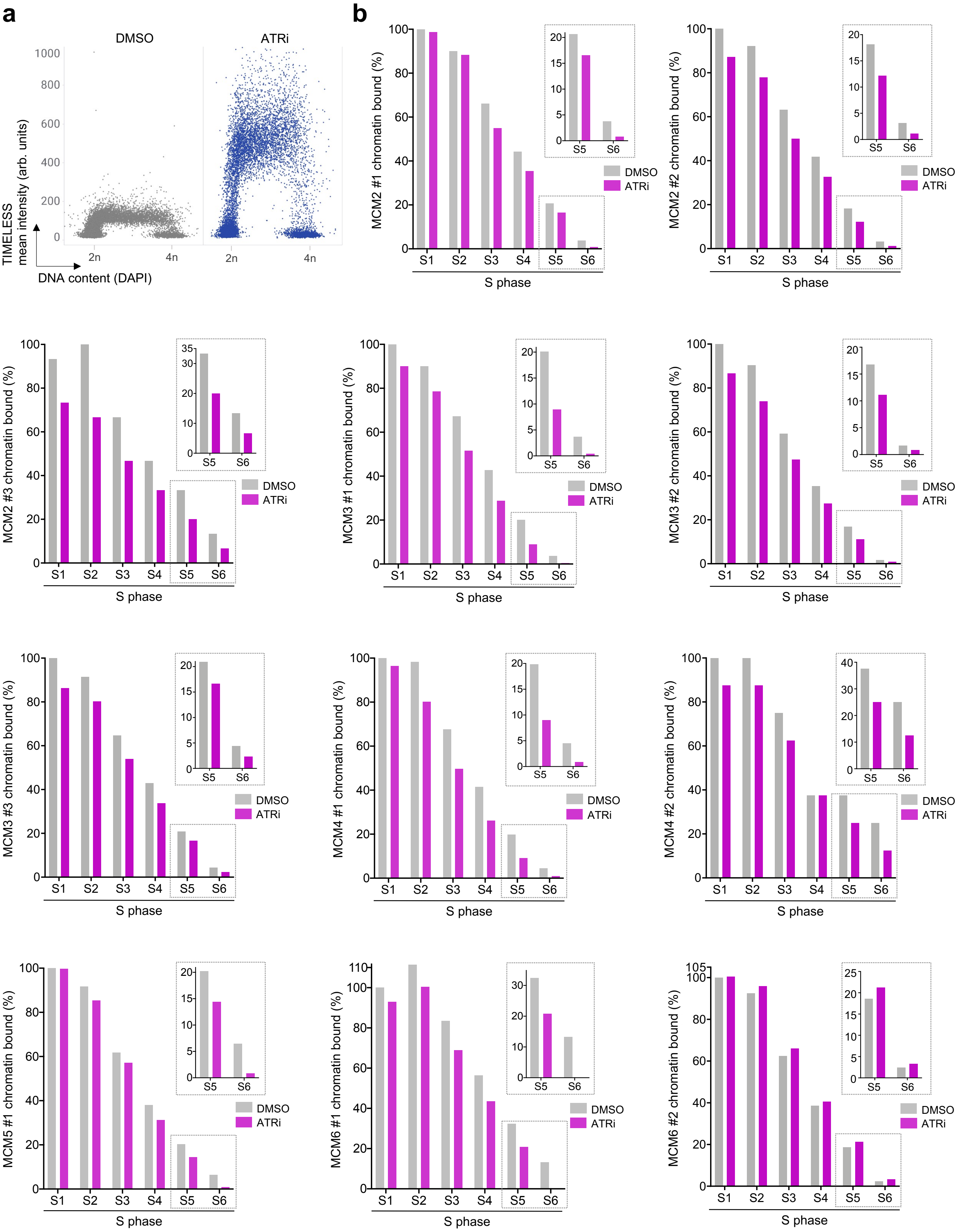

a
b
DMSO
ATRi
TIMELESS mean intensity (arb. units)
DNA content (DAPI)
2n
4n
MCM2 #1 chromatin bound (%)
MCM2 #2 chromatin bound (%)
MCM2 #3 chromatin bound (%)
MCM3 #1 chromatin bound (%)
MCM3 #2 chromatin bound (%)
MCM3 #3 chromatin bound (%)
MCM4 #1 chromatin bound (%)
MCM4 #2 chromatin bound (%)
MCM5 #1 chromatin bound (%)
MCM6 #1 chromatin bound (%)
MCM6 #2 chromatin bound (%)
S1
S2
S3
S4
S5
S6
S phase

Supplementary Fig. 4 | Immunostained MCMs display decreased localization in RFs upon increased origin firing. a, QIBC of U2OS cells treated with ATR inhibitor (ATRi) and immunostained for chromatin-bound TIMELESS. Nuclear DNA was counterstained by DAPI; n ≈10,000 cells per condition; arb. (arbitrary) units. **b,** QIBC-based analysis of chromatin-bound MCMs detected by indicated antibodies at different intervals of S phase sub-stratified as in (Supplementary Fig. 1b) in the presence of DMSO or ATR inhibitor (ATRi). Each bar indicates median of mean intensity normalized to 100 percent with respect to MCM intensity in the S1 stage of S phase of the untreated cells; n ≈10,000 cells per condition. Source data are provided as a Source data file.

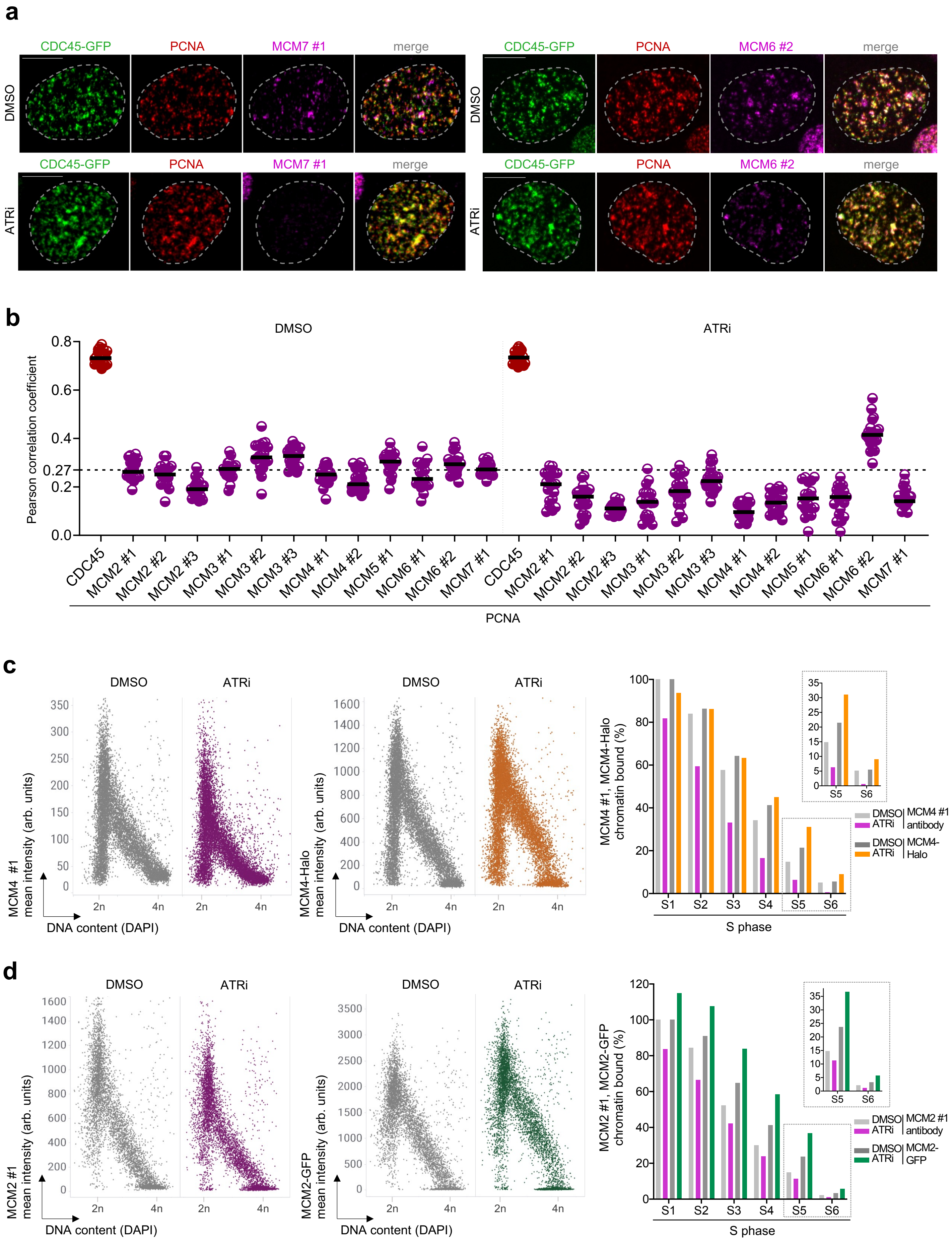
a
CDC45-GFP
PCNA
MCM7 #1
merge
CDC45-GFP
PCNA
MCM6 #2
merge
DMSO
ATRi
b
DMSO
ATRi
Pearson correlation coefficient
0.8
0.6
0.4
0.27
0.2
0.0
CDC45
MCM2 #1
MCM2 #2
MCM2 #3
MCM3 #1
MCM3 #2
MCM3 #3
MCM4 #1
MCM4 #2
MCM5 #1
MCM6 #1
MCM6 #2
MCM7 #1
PCNA
c
DMSO
ATRi
MCM4 #1 mean intensity (arb. units)
DNA content (DAPI)
2n
4n
MCM4-Halo mean intensity (arb. units)
MCM4 #1, MCM4-Halo chromatin bound (%)
S1
S2
S3
S4
S5
S6
S phase
DMSO
ATRi
MCM4 #1 antibody
MCM4-Halo
d
DMSO
ATRi
MCM2 #1 mean intensity (arb. units)
DNA content (DAPI)
MCM2-GFP mean intensity (arb. units)
MCM2 #1, MCM2-GFP chromatin bound (%)
S phase
MCM2 #1 antibody
MCM2-GFP

Supplementary Fig.5 | Immunostained and endogenously tagged MCMs show opposite chromatin binding dynamics during S phase upon increased origin firing. a, Left and right, representative MIP confocal images of chromatin-bound CDC45-GFP, PCNA and MCM7 (in left panel) or MCM6 (in right panel) detected by indicated antibodies in the S5 stage of S phase (see Supplementary Fig. 1b for S phase sub-stratification) under normal conditions or after ATR inhibitor (ATRi) treatment. Scale bar, 10 μm. **b,** Colocalization analysis of immunostained MCMs with RFs marked by immunostained PCNA in the S5 stage of S phase in control conditions (DMSO) or after ATR inhibitor (ATRi) treatment. Horizontal lines are medians; n = 20 cells per condition. The dashed horizontal line represents the average of the Pearson correlation coefficient for MCMs and CDC45-GFP colocalization in DMSO treatment. **c,** Left and middle, QIBC of CDC45-GFP cells with endogenously tagged MCM4-Halo treated with ATR inhibitor and pulsed with JF549 HaloTag ligand. Cells were additionally immunostained for chromatin-bound MCM4; arb. (arbitrary) units. Right, quantification of QIBC plots (left and middle) at different intervals of S phase sub-stratified as in (Supplementary Fig. 1b). Each bar indicates median of mean intensity normalized to 100 percent with respect to immunostained MCM4 or endogenously tagged MCM4-Halo intensities in the S1 stage of S phase in untreated cells; n ≈10,000 cells per condition. **d,** Left and middle, QIBC of MCM2-GFP cells treated with ATR inhibitor and immunostained for chromatin-bound GFP, PCNA and MCM2. Nuclear DNA was counterstained by DAPI; n ≈6,000 cells per condition; arb. (arbitrary) units. Right, quantification of QIBC plots (left and middle) at different intervals of S phase sub-stratified as in (Supplementary Fig. 1b). Each bar indicates median of mean intensity normalized to 100 percent with respect to immunostained MCM2 or endogenously tagged MCM2-GFP intensities in the S1 stage of S phase in the untreated cells; n ≈6,000 cells per condition. Source data are provided as a Source data file.

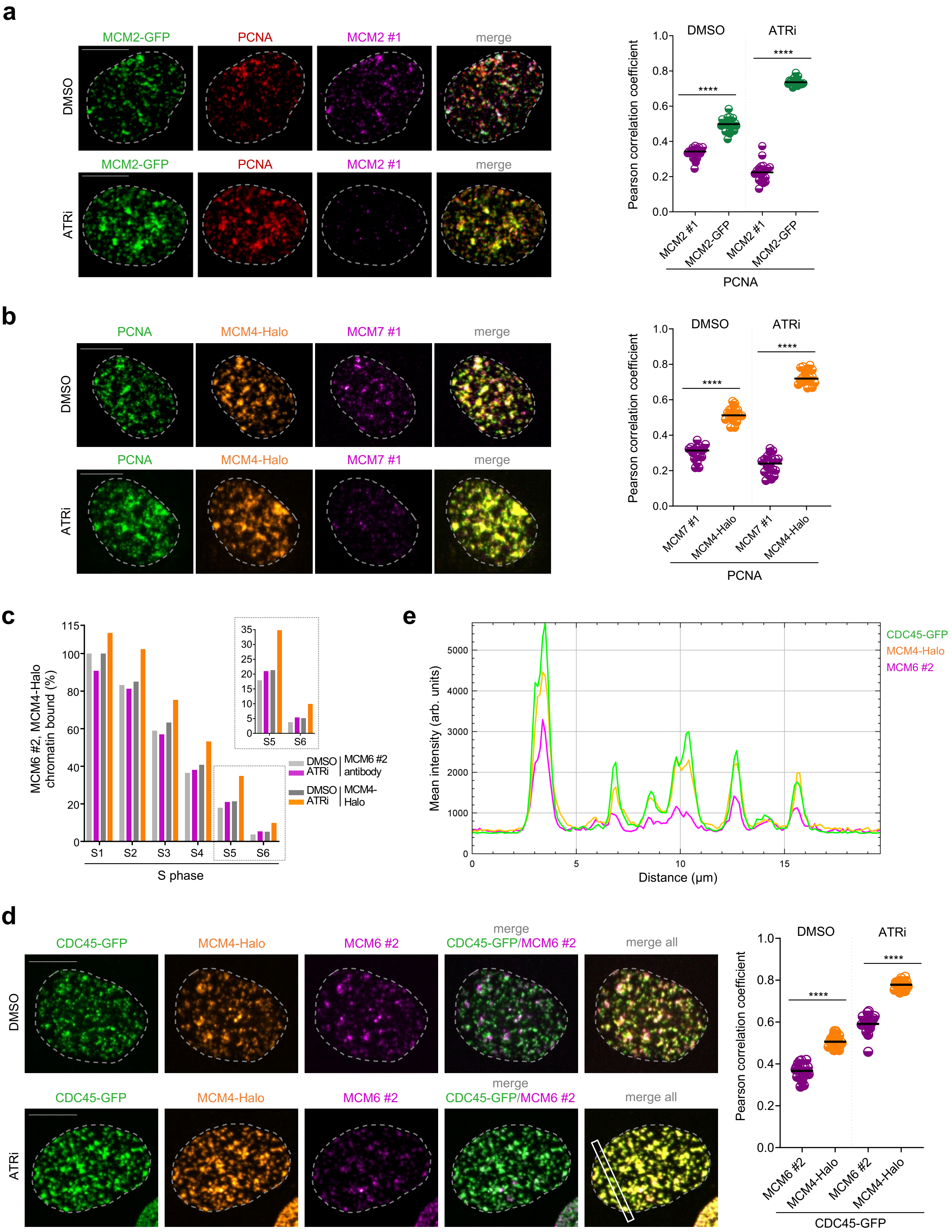
a
MCM2-GFP
PCNA
MCM2 #1
merge
DMSO
ATRi
Pearson correlation coefficient
DMSO
ATRi
****
****
1.0
0.8
0.6
0.4
0.2
0.0
MCM2 #1
MCM2-GFP
MCM2 #1
MCM2-GFP
PCNA
b
PCNA
MCM4-Halo
MCM7 #1
merge
DMSO
ATRi
Pearson correlation coefficient
DMSO
ATRi
****
****
1.0
0.8
0.6
0.4
0.2
0.0
MCM7 #1
MCM4-Halo
MCM7 #1
MCM4-Halo
PCNA
c
MCM6 #2, MCM4-Halo chromatin bound (%)
115
100
80
60
40
20
0
S1
S2
S3
S4
S5
S6
S phase
35
30
25
20
15
10
5
0
S5
S6
DMSO
ATRi
MCM6 #2 antibody
DMSO
ATRi
MCM4-Halo
e
Mean intensity (arb. units)
5000
4000
3000
2000
1000
0
0
5
10
15
Distance (μm)
CDC45-GFP
MCM4-Halo
MCM6 #2
d
CDC45-GFP
MCM4-Halo
MCM6 #2
merge
CDC45-GFP/MCM6 #2
merge all
DMSO
ATRi
Pearson correlation coefficient
DMSO
ATRi
****
****
1.0
0.8
0.6
0.4
0.2
0.0
MCM6 #2
MCM4-Halo
MCM6 #2
MCM4-Halo
CDC45-GFP

Supplementary Fig. 6 | MCM6 #2 antibody partially detects active MCMs in RFs. a, Left, representative MIP confocal images of chromatin-bound MCM2-GFP, PCNA and immunostained MCM2 in the S5 stage of S phase during normal conditions or ATR inhibitor (ATRi) treatment. Scale bar, 10 µm. Right, colocalization analysis of MCM2-GFP and immunostained MCM2 with RFs marked by PCNA in the S5 stage of S phase. Horizontal lines are medians; n = 20 cells per condition; *P* values were calculated by two-tailed unpaired t-test; $^{****}P > 0.0001$. **b,** Left, representative MIP confocal images of RPE cells with chromatin-bound MCM4-Halo and immunostained PCNA and MCM7 in the S5 stage of S phase under indicated treatment. Scale bar, 10 µm. Right, colocalization analysis of RPE cells with MCM4-Halo and immunostained MCM7 with RFs marked by PCNA in the S5 stage of S phase under indicated treatment. Horizontal lines are medians; n = 20 cells per condition; *P* values were calculated by two-tailed unpaired t-test; $^{****}P > 0.0001$. **c,** QIBC-based analysis of chromatin-bound MCM4-Halo and immunostained MCM6 at different intervals of S phase under indicated treatment. Each bar indicates median of mean intensity normalized to 100 percent with respect to immunostained MCM6 or MCM4-Halo intensities in the S1 stage of S phase in untreated cells; n ≈10,000 cells per condition. **d,** Left, representative MIP confocal images of chromatin-bound CDC45-GFP, MCM4-Halo and immunostained MCM6 in the S5 stage of S phase under indicated treatment. Scale bar, 10 µm. Right, colocalization analysis of MCM4-Halo and immunostained MCM7 with RFs marked by CDC45-GFP in the S5 stage of S phase under indicated treatment. Horizontal lines are medians; n = 20 cells per condition; *P* values were calculated by two-tailed unpaired t-test; $^{****}P > 0.0001$. **e,** Profile plot of RFs indicated by white box in (**d**) and visualized by CDC45-GFP, MCM4-Halo, and MCM6 antibody. See Supplementary Fig. 1b for S phase sub-stratification. Source data are provided as a Source data file.

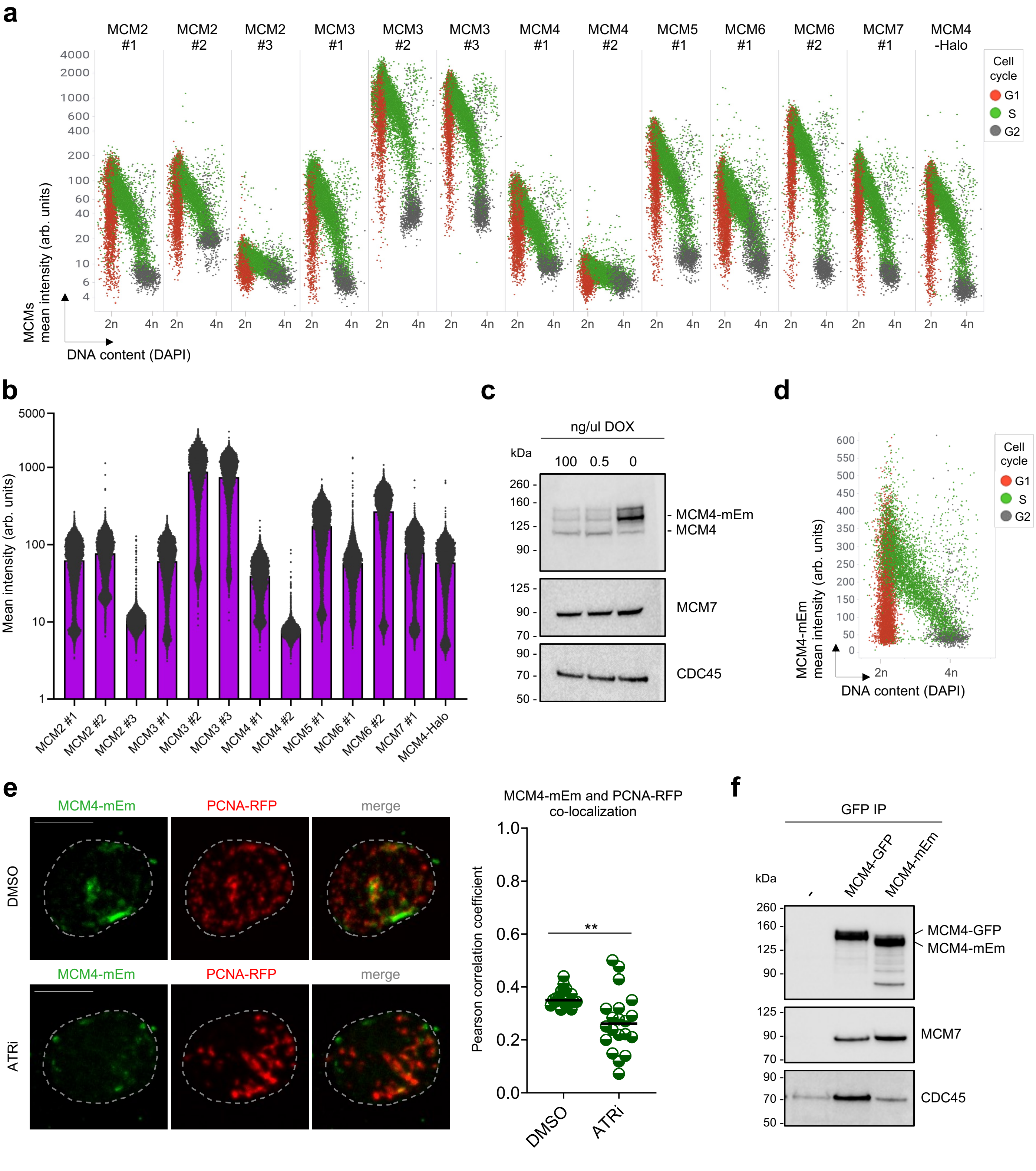
a
MCM2 #1
MCM2 #2
MCM2 #3
MCM3 #1
MCM3 #2
MCM3 #3
MCM4 #1
MCM4 #2
MCM5 #1
MCM6 #1
MCM6 #2
MCM7 #1
MCM4 -Halo
Cell cycle
G1
S
G2
MCMs mean intensity (arb. units)
DNA content (DAPI)
2n
4n
b
Mean intensity (arb. units)
MCM2 #1
MCM2 #2
MCM2 #3
MCM3 #1
MCM3 #2
MCM3 #3
MCM4 #1
MCM4 #2
MCM5 #1
MCM6 #1
MCM6 #2
MCM7 #1
MCM4-Halo
c
ng/ul DOX
kDa
100
0.5
0
MCM4-mEm
MCM4
MCM7
CDC45
d
Cell cycle
G1
S
G2
MCM4-mEm mean intensity (arb. units)
DNA content (DAPI)
e
MCM4-mEm
PCNA-RFP
merge
DMSO
ATRi
MCM4-mEm and PCNA-RFP co-localization
Pearson correlation coefficient
**
DMSO
ATRi
f
GFP IP
kDa
-
MCM4-GFP
MCM4-mEm
MCM4-GFP
MCM4-mEm
MCM7
CDC45

Supplementary Fig. 7 | Ectopic overexpression of MCMs without removing the endogenous levels leads to inefficient CMG formation. a, QIBC of CDC45-GFP cells immunostained for chromatin-bound PCNA and the indicated MCMs acquired under the same imaging conditions. Nuclear DNA was counterstained by DAPI. PCNA was used as an additional cell cycle marker; n ≈10,000 cells per condition; arb. (arbitrary) units. **b,** Quantification of QIBC plots in (**a**). Each bar indicates median of mean intensity; n ≈10,000 cells per condition; arb. (arbitrary) units. **c,** Immunoblotting of total cell extracts from CHO cells ectopically expressing MCM4-mEmerald (mEm) under tet-off promoter regulated by indicated doxycycline concentrations for 24 hours. n = 3 biological replicates. **d,** QIBC of CHO cells ectopically expressing MCM4-mEmerald and PCNA-RFP. Nuclear DNA was counterstained by DAPI. PCNA-RFP was used as a cell cycle marker; n ≈10,000 cells per condition; arb. (arbitrary) units. **e,** Left, representative MIP confocal images of CHO cells with chromatin-bound MCM4-mEmerald and PCNA-RFP in the S5 stage of S phase (see Supplementary Fig. 1b for S phase sub-stratification) under normal conditions or after ATR inhibitor (ATRi) treatment. Scale bar, 10 µm. Right, colocalization analysis of CHO cells ectopically expressing MCM4-mEmerald and RFs marked by PCNA-RFP in the S5 stage of S phase under indicated treatment. Horizontal lines are medians; n = 20 cells per condition; *P* value was determined by two-tailed unpaired t-test; ***P* = 0.0013. **f,** GFP-immunoprecipitation (GFP IP) followed by immunoblotting of total cell extracts from U2OS cells with endogenously tagged MCM4-GFP or CHO cells ectopically expressing MCM4-mEmerald without doxycycline treatment for 24 hours. Immunoprecipitants were adjusted to the same level of MCM4. n = 3 biological replicates. Source data are provided as a Source data file.

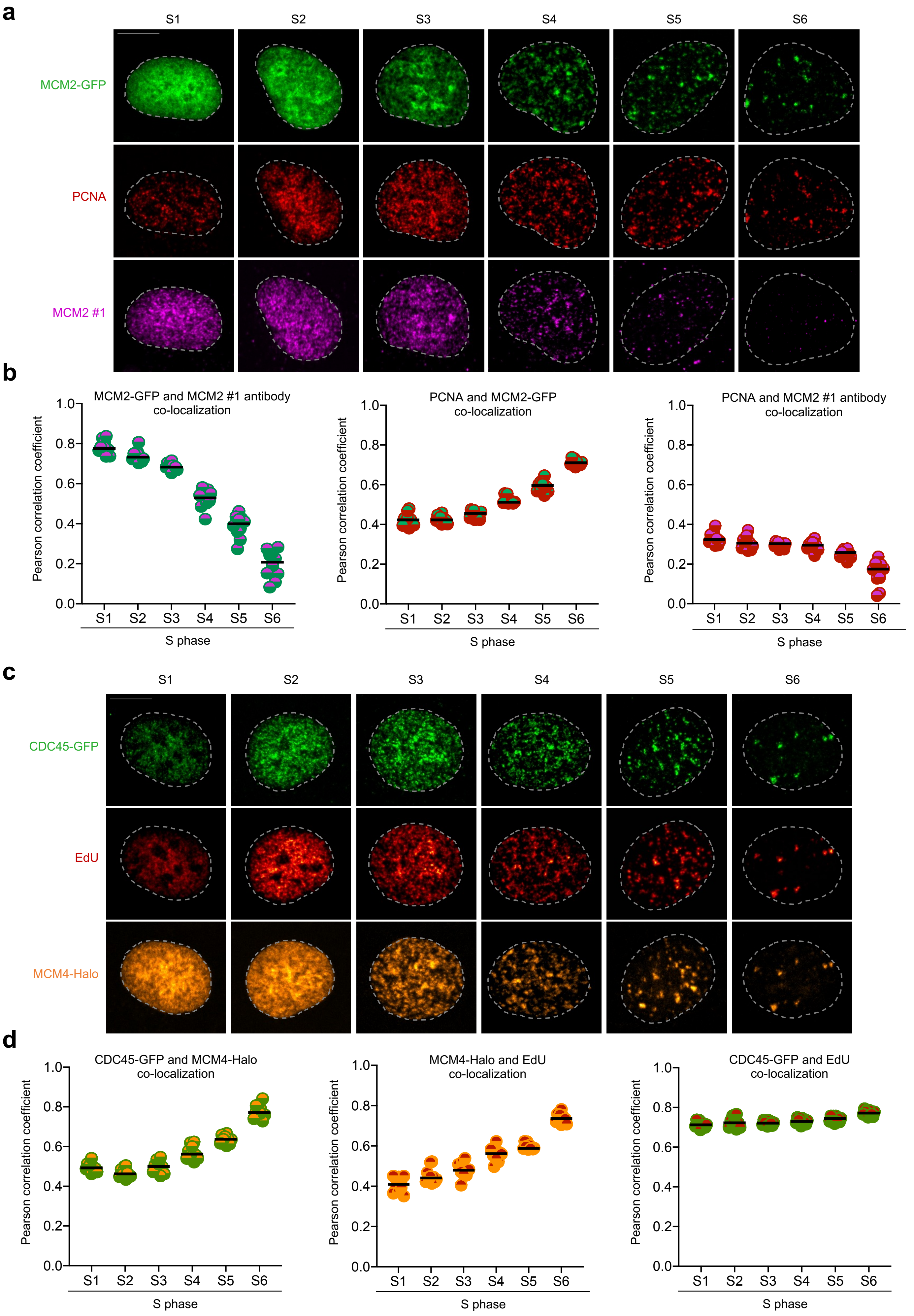

a
S1
S2
S3
S4
S5
S6
MCM2-GFP
PCNA
MCM2 #1
b
MCM2-GFP and MCM2 #1 antibody co-localization
PCNA and MCM2-GFP co-localization
PCNA and MCM2 #1 antibody co-localization
Pearson correlation coefficient
1.0
0.8
0.6
0.4
0.2
0.0
S phase
c
CDC45-GFP
EdU
MCM4-Halo
d
CDC45-GFP and MCM4-Halo co-localization
MCM4-Halo and EdU co-localization
CDC45-GFP and EdU co-localization

Supplementary Fig. 8 | Endogenous tagging of MCM subunits enables visualization of the MCM scaffold at active replisomes. a, Representative MIP confocal images of chromatin-bound MCM2-GFP, PCNA, and MCM2 detected by antibody at the indicated S phase sub-stratified as in (Supplementary Fig. 1b). Scale bar, 10 µm. **b,** Left, colocalization analysis of MCM2-GFP and immunostained MCM2 at the indicated S phase stages. Middle, colocalization analysis of PCNA and MCM2-GFP at the indicated S phase stages. Right, colocalization analysis of PCNA and immunostained MCM2 at the indicated S phase stages. Horizontal lines are medians; n = 10 cells per condition. **c,** Representative MIP confocal images of chromatin-bound CDC45-GFP, MCM4-Halo and EdU during S phase sub-stratified as in (Supplementary Fig. 1b). Scale bar, 10 µm. **d,** Left, colocalization analysis of CDC45-GFP and MCM4-Halo at the indicated S phase stages. Middle, colocalization analysis of MCM4-Halo and EdU at the indicated S phase stages. Right, colocalization analysis of CDC45-GFP and EdU at the indicated S phase stages. Horizontal lines are medians; n = 10 cells per condition. Source data are provided as a Source data file.

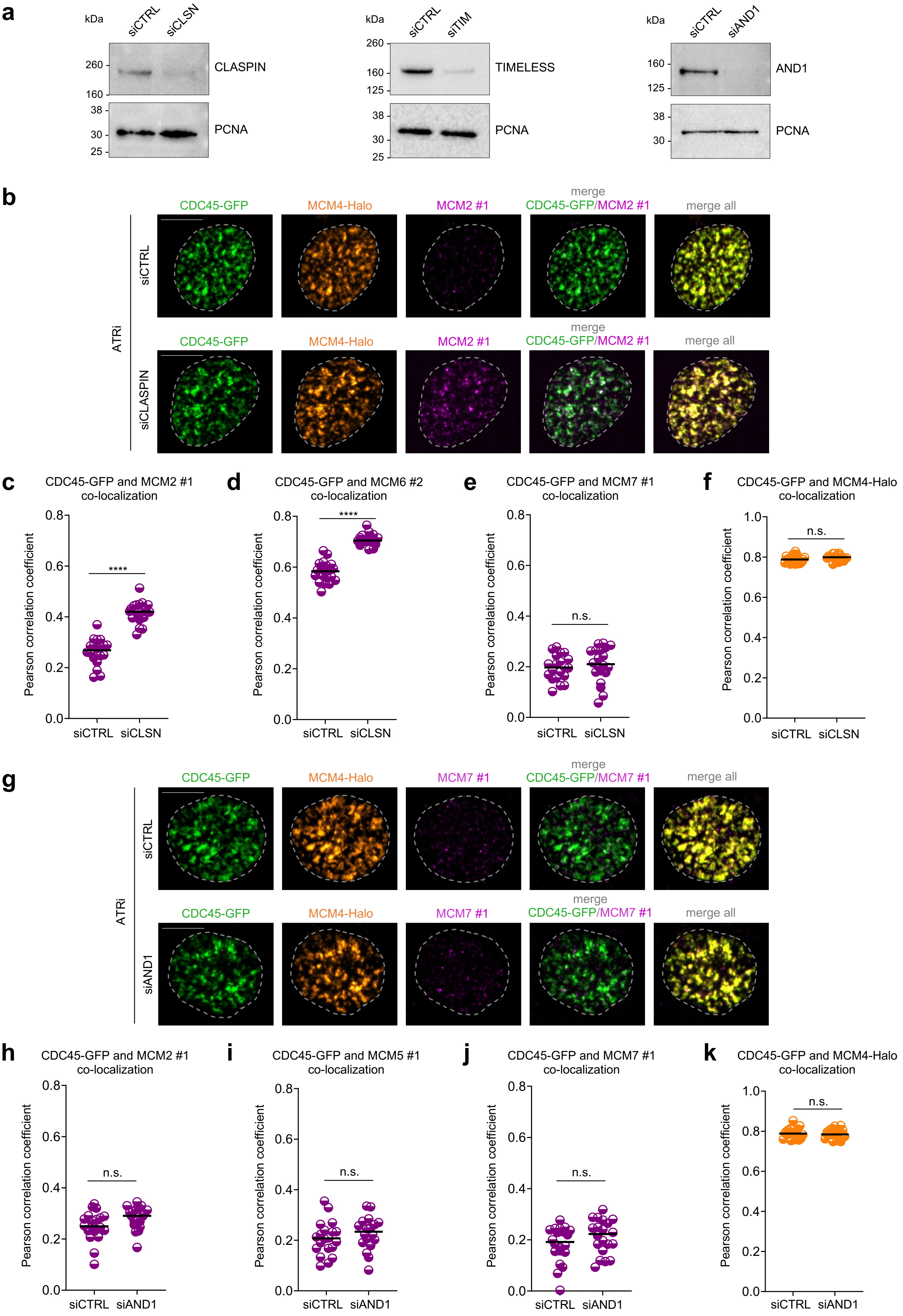
a
kDa
siCTRL
siCLSN
260
160
CLASPIN
38
30
25
PCNA
siTIM
125
TIMELESS
siAND1
AND1
b
CDC45-GFP
MCM4-Halo
MCM2 #1
merge
CDC45-GFP/MCM2 #1
merge all
ATRi
siCLASPIN
c
CDC45-GFP and MCM2 #1 co-localization
Pearson correlation coefficient
****
d
CDC45-GFP and MCM6 #2 co-localization
e
CDC45-GFP and MCM7 #1 co-localization
n.s.
f
CDC45-GFP and MCM4-Halo co-localization
g
MCM7 #1
CDC45-GFP/MCM7 #1
h
i
CDC45-GFP and MCM5 #1 co-localization
j
k

Supplementary Fig. 9 | CLASPIN, but not AND-1, interaction with CMG restricts the detection of active MCMs at fully assembled replication forks. a, Western blot of CDC45-GFP, MCM4-Halo cell line treated with control siRNA and siRNA against *CLASPIN* (abbreviated as CLSN), *TIMELESS* (abbreviated as TIM) and *AND1* (5 nM; 24 h) and ATR inhibitor; n = 2 biological replicates. **b,** Representative MIP confocal images of chromatin-bound CDC45-GFP, MCM4-Halo and immunostained MCM2 in S5 stage of S phase sub-stratified as in (Supplementary Fig. 1b). Cells were transfected by control siRNA or siRNA against *CLASPIN* as indicated and treated with ATR inhibitor (ATRi). Scale bar, 10 µm. **c,** Colocalization of CDC45-GFP and immunostained MCM2 in the S5 stage of S phase. **d,** Colocalization of CDC45-GFP and immunostained MCM6 in the S5 stage of S phase. **e,** Colocalization of CDC45-GFP and immunostained MCM7 in the S5 stage of S phase. **f,** Colocalization of CDC45-GFP and MCM4-Halo in the S5 stage of S phase. Cells in (**c-f**) were treated as in (**b**). Lines in (**c-f**) are medians; n = 20 cells per condition; *P* values in (**c-f**) were calculated by two-tailed unpaired t-test; not significant (n.s.) denotes $P > 0.05$; ****$P > 0.0001$. **g,** Representative MIP confocal images of chromatin-bound CDC45-GFP, MCM4-Halo and immunostained MCM2 in S5 stage of S phase sub-stratified as in (Supplementary Fig. 1b). Cells were transfected by control siRNA or siRNA against *AND-1* as indicated and treated with ATR inhibitor (ATRi). Scale bar, 10 µm. **h,** Colocalization of CDC45-GFP and immunostained MCM2 in the S5 stage of S phase. **i,** Colocalization of CDC45-GFP and immunostained MCM5 in the S5 stage of S phase. **j,** Colocalization of CDC45-GFP and immunostained MCM7 in the S5 stage of S phase. **k,** Colocalization of CDC45-GFP and MCM4-Halo in the S5 stage of S phase. Cells in (**h-k**) were treated as in (**g**). Lines in (**h-k**) are medians; n = 20 cells per condition; *P* values in (**h-k**) were calculated by two-tailed unpaired t-test; not significant (n.s.) denotes $P > 0.05$. Source data are provided as a Source data file.
