# Supplementary material for: Solving the MCM paradox by visualizing the scaffold of CMG helicase at active replisomes
Source: Nat Commun. 2022 Oct 14;13:6090. doi: 10.1038/s41467-022-33887-5 (PMC9568601; doi:10.1038/s41467-022-33887-5)
Supplement: Supplementary file 3 — Reporting Summary [file 41467_2022_33887_MOESM3_ESM.pdf]

nature portfolio

Corresponding author(s): Hana Polasek-Sedlackova, Jiri Lukas

Last updated by author(s): Sep 29, 2022

Reporting Summary

Nature Portfolio wishes to improve the reproducibility of the work that we publish. This form provides structure for consistency and transparency in reporting. For further information on Nature Portfolio policies, see our Editorial Policies and the Editorial Policy Checklist.

Statistics

For all statistical analyses, confirm that the following items are present in the figure legend, table legend, main text, or Methods section.

n/a	Confirmed	
☐	☒	The exact sample size (*n*) for each experimental group/condition, given as a discrete number and unit of measurement
☐	☒	A statement on whether measurements were taken from distinct samples or whether the same sample was measured repeatedly
☐	☒	The statistical test(s) used AND whether they are one- or two-sided<br>*Only common tests should be described solely by name; describe more complex techniques in the Methods section.*
☒	☐	A description of all covariates tested
☒	☐	A description of any assumptions or corrections, such as tests of normality and adjustment for multiple comparisons
☐	☒	A full description of the statistical parameters including central tendency (e.g. means) or other basic estimates (e.g. regression coefficient) AND variation (e.g. standard deviation) or associated estimates of uncertainty (e.g. confidence intervals)
☐	☒	For null hypothesis testing, the test statistic (e.g. *F*, *t*, *r*) with confidence intervals, effect sizes, degrees of freedom and *P* value noted<br>*Give P values as exact values whenever suitable.*
☒	☐	For Bayesian analysis, information on the choice of priors and Markov chain Monte Carlo settings
☒	☐	For hierarchical and complex designs, identification of the appropriate level for tests and full reporting of outcomes
☐	☒	Estimates of effect sizes (e.g. Cohen's *d*, Pearson's *r*), indicating how they were calculated

Our web collection on statistics for biologists contains articles on many of the points above.

Software and code

Policy information about availability of computer code

Data collection	ScanR acquisition software (Olympus, v.2.7.1 for ScanR high-content microscopy)<br>Volocity acquisition software (Perkin Elmer, v.6.3, for Ultraview Vox spinning disk microscopy)
Data analysis	ScanR Analysis (Olympus, v.2.7.1, for QIBC)<br>Excel (Microsoft, v.16.57, for image analysis data management)<br>Spotfire (Tibco, v.10.5.0.72, for data visualization)<br>Volocity (Perkin Elmer, v.6.3, for co-localization analysis and image viewing)<br>GraphPad Prism (v.9.0.0, for data visualization and statistics)<br>PyMOL Molecular Graphics System (Schrödinger, LLC, v.2.5.2, for structural analysis)

For manuscripts utilizing custom algorithms or software that are central to the research but not yet described in published literature, software must be made available to editors and reviewers. We strongly encourage code deposition in a community repository (e.g. GitHub). See the Nature Portfolio guidelines for submitting code & software for further information.

Data

Policy information about availability of data

All manuscripts must include a data availability statement. This statement should provide the following information, where applicable:

- Accession codes, unique identifiers, or web links for publicly available datasets
- A description of any restrictions on data availability
- For clinical datasets or third party data, please ensure that the statement adheres to our policy

Source data are provided with this paper. Primary imaging data have been deposited at the European Bioinformatics Institute (EBI) BioStudies database under accession code S-BSST909 [https://www.ebi.ac.uk/biostudies/studies/S-BSST909?key=9594f0c6-c01c-4faf-954b-b08f58075dfd]. The electron microscopy data for S. cerevisiae MCM2-7 double hexamers used in this study are available in the Protein Data Bank database under accession code 6F0L [https://www.rcsb.org/structure/6F0L]. The electron microscopy data for human core leading strand replisome used in this study are available in the Protein Data Bank database under accession code 7PFO [https://www.rcsb.org/structure/7PFO]. Any additional data or information in support of this study will be available from corresponding authors upon reasonable request.

Human research participants

Policy information about studies involving human research participants and Sex and Gender in Research.

Reporting on sex and gender	Not applicable, because this study does not involve human research participants.
Population characteristics	Not applicable, because this study does not involve human research participants.
Recruitment	Not applicable, because this study does not involve human research participants.
Ethics oversight	Not applicable, because this study does not involve human research participants.

Note that full information on the approval of the study protocol must also be provided in the manuscript.

Field-specific reporting

Please select the one below that is the best fit for your research. If you are not sure, read the appropriate sections before making your selection.

☒ Life sciences ☐ Behavioural & social sciences ☐ Ecological, evolutionary & environmental sciences

For a reference copy of the document with all sections, see nature.com/documents/nr-reporting-summary-flat.pdf

Life sciences study design

All studies must disclose on these points even when the disclosure is negative.

Sample size	No statistical method was used to predetermine the sample size. For QIBC at least 5000 cells per condition were acquired, which was determined based on current standards in the high-content imaging field (e.g. Toledo et al., Cell 2013 Nov 21;155(5):1088-103.; Somyajit et al, Science 2017 Nov 10;358(6364):797-802.; Sedlackova et al., Nature, 2020 Oct 21; 587, 297-302). For spinning disc confocal microscopy, 10 to 40 cells per condition were acquired and the statistical power was evidenced by conditions with a p-value of t-test <0.0001.
Data exclusions	All data acquired for this study were included in the analysis.
Replication	All experimental findings were reliably reproduced as indicated in the figure legends. All attempts of replication were successful.
Randomization	No randomization was done, because this study does not involve animals or human participants. Samples were organized into groups based on treatments (e.g. untreated vs. treatment; control siRNA treated compared to target-specific siRNAs; experimental time-points). Appropriate controls were included in all experiments.
Blinding	For QIBC, data collection and data analysis were conducted using automated image acquisition and analysis software, therefore blinding was not necessarily due to the intrinsically unbiased nature of the approach. For spinning disc confocal microscopy, the data acquisition was organized in a way to capture images from the overall sample in an unbiased manner, and analysis was carried out using automated analysis. No animal or human research participants or samples were involved, making additional levels of blinding unnecessary.

Reporting for specific materials, systems and methods

We require information from authors about some types of materials, experimental systems and methods used in many studies. Here, indicate whether each material, system or method listed is relevant to your study. If you are not sure if a list item applies to your research, read the appropriate section before selecting a response.

Materials & experimental systems

n/a	Involved in the study
☐	☒ Antibodies
☐	☒ Eukaryotic cell lines
☒	☐ Palaeontology and archaeology
☒	☐ Animals and other organisms
☒	☐ Clinical data
☒	☐ Dual use research of concern

Methods

n/a	Involved in the study
☒	☐ ChIP-seq
☒	☐ Flow cytometry
☒	☐ MRI-based neuroimaging

Antibodies

Antibodies used

Primary antibodies for immunofluorescence (IF) were used as follows: GFP (rabbit, Chromotek, PABG1-100, 1:1,000), MCM2 #1 (mouse, Novus Biologicals, H00004171-M01, clone 6A8, 1:1,000), MCM2 #2 (rabbit, Proteintech, 10513-1-AP, 1:1,000), MCM2 #3 (mouse, Santa Cruz, sc-373702, clone E-8, 1:1,000), MCM3 #1 (mouse, Santa Cruz, sc-390480, clone E-8, 1:1,000), MCM3 #2 (rabbit, Antibodies.com, A29136, 1:1,000), MCM3 #3 (rabbit, Abcam, ab4460, 1:1,000), MCM4 #1 (mouse, Novus Biologicals, H00004173-B01P, 1:1,000), MCM4 #2 (mouse, Santa Cruz, sc-28317, clone G-7, 1:1,000), MCM5 #1 (rabbit, Abcam, ab17967, 1:1,000), MCM6 #1 (rabbit, Abcam, ab201683, clone EPR17686, 1:1,000), MCM6 #2 (mouse, Novus Biologicals, H00004175-M04, clone 7D8, 1:1,000), MCM7 #1 (mouse, Santa Cruz, sc-9966, clone 141.2, 1:1,000), PCNA (human, Immuno Concepts, 2037, 1:1,000), TIMELESS (rabbit, Abcam, ab109512, clone EPR5275, 1:500).

Primary antibody for western blotting (WB) were used as follows: AND-1 (rabbit, Abcam, ab224221, 1:1,000), CDC45 (rabbit, Cell Signaling Technology, 11881S, clone D7G6, 1:1,000), CLASPIN (mouse, Santa Cruz, sc-376773, clone B-6, 1:1,000), GFP (rabbit, Chromotek, PABG1-100, 1:1,000), KAP-1 (rabbit, Bethyl Laboratories, A300-274A, 1:2,000), MCM4 #1 (mouse, Novus Biologicals, H00004173-B01P, 1:1000), MCM7 #1 (mouse, monoclonal, Santa Cruz, sc-9966, clone 141.2, 1:1000), PCNA (mouse, Santa Cruz, sc-56, clone PC-10, 1:1,000), POLD1 (rabbit, Abcam, ab186407, clone EPR15118, 1:1,000), POLE1 (rabbit, Abcam, ab226848, 1:1,000), TIMELESS (rabbit, Abcam, ab109512, clone EPR5275, 1:1,000).

Secondary antibody conjugates for IF were goat anti-mouse and goat anti rabbit Alexa Fluor 488 (A11029, A11034), Alexa Fluor 568 (A11031, A11036) Alexa Fluor 647 (A21236, A21245) (all from Thermo Fischer Scientific, 1:1000) and donkey anti-human Alexa Fluor 647 (Jackson Immuno Research, 709-605-149, 1:1000).

Secondary antibody conjugates for WB were HRP horse anti-mouse IgG antibody (Vector Laboratories, PI-2000, 1:10000) and HRP goat anti-rabbit IgG antibody (Vector Laboratories, PI-1000, 1:10000).

Validation

AND-1 (rabbit, Abcam, ab224221, 1:1,000) was previously validated by the manufacturer in the western blot experiment detecting AND1 expression in whole cell lysate samples of various cell lines and it was further validated in this study (Supplementary Fig. 9a) by the western blot experiment detecting AND1 expression in whole cell lysates of U2OS cells treated with control siRNA or siRNA against AND1.

CDC45 (rabbit, Cell Signaling Technology, 11881S, clone D7G6, 1:1,000) was previously validated by the manufacturer in the western blot experiment detecting CDC45 expression in whole cell lysate samples of various cell lines and it was further validated in this study (Supplementary Fig. 1a) by western blot of naïve U2OS cells and U2OS cell line with GFP-tagged CDC45.

CLASPIN (mouse, Santa Cruz, sc-376773, clone B-6, 1:1,000) was previously validated by the manufacturer in the western blot experiment detecting CLASPIN expression in whole cell lysate samples of various cell lines and it was further validated in this study (Supplementary Fig. 9a) by the western blot experiment detecting CLASPIN expression in whole cell lysates of U2OS cells treated with control siRNA or siRNA against CLASPIN.

GFP (rabbit, Chromotek, PABG1-100, 1:1,000) was previously validated by the manufacturer in the western blot experiment detecting purified recombinant GFP protein in concentrations ranging from 0.8 ng to 25 ng and it was further validated in this study by western blot of naïve U2OS cells and cell lines with GFP-tagged replisome components (Supplementary Fig. 1a, d, e).

KAP-1 (rabbit, Bethyl Laboratories, A300-274A, 1:2,000) was previously validated by the manufacturer in the western blot experiment detecting KAP-1 immunoprecipitated from HeLa cells and it was further validated by western blot experiments in previous studies e.g. Somyajit et al, Science 2017 Nov 10;358(6364):797-802.; Spies et al, Nat Cell Biol. 2019 Apr;21(4):487-497.

MCM2 #1 (mouse, Novus Biologicals, H00004171-M01, clone 6A8, 1:1,000) was previously validated by the manufacturer in the western blot experiment detecting MCM2 in transfected and un-transfected lysates of 293T cell line and it was further validated for immunofluorescence experiments in (Sedlackova et al., Nature, 2020 Oct 21; 587, 297-302).

MCM2 #2 (rabbit, Proteintech, 10513-1-AP, 1:1,000) was previously validated by the manufacturer in the western blot experiment detecting MCM2 in control and MCM2 depleted HEK-293 cells and it was further validated for immunofluorescence experiments by QIBC in this study (Supplementary Fig. 2 and 4).

MCM2 #3 (mouse, Santa Cruz, sc-373702, clone E-8, 1:1,000) was previously validated by the manufacturer in the western blot experiment detecting MCM2 expression in HeLa and Jurkat whole cell lysates and it was further validated for immunofluorescence

experiments by QIBC in this study (Supplementary Fig. 2 and 4).

MCM3 #1 (mouse, Santa Cruz, sc-390480, clone E-8, 1:1,000) was previously validated by the manufacturer in the western blot experiment detecting MCM3 expression in whole cell lysate samples of various cell lines and it was further validated for immunofluorescence experiments in (Sedlackova et al., Nature, 2020 Oct 21; 587, 297-302).

MCM3 #2 (rabbit, Antibodies.com, A29136, 1:1,000) was previously validated by the manufacturer in the western blot experiment detecting MCM3 expression in whole cell lysate samples of various cell lines and it was further validated for immunofluorescence experiments by QIBC in this study (Supplementary Fig. 2 and 4).

MCM3 #3 (rabbit, Abcam, ab4460, 1:1,000) was previously validated by the manufacturer in the western blot experiment detecting MCM3 immunoprecipitated from HeLa cells and it was further validated for immunofluorescence experiments by QIBC in this study (Supplementary Fig. 2 and 4).

MCM4 #1 (mouse, Novus Biologicals, H00004173-B01P, 1:1,000) was previously validated by the manufacturer in the western blot experiment detecting MCM4 in transfected and un-transfected lysates of 293T cell line and it was further validated for immunofluorescence experiments in (Sedlackova et al., Nature, 2020 Oct 21; 587, 297-302).

MCM4 #2 (mouse, Santa Cruz, sc-28317, clone G-7, 1:1,000) was previously validated by the manufacturer in the western blot experiment detecting MCM4 expression in HeLa whole cell lysates and it was further validated for immunofluorescence experiments by QIBC in this study (Supplementary Fig. 2 and 4).

MCM5 #1 (rabbit, Abcam, ab17967, 1:1,000) was previously validated by the manufacturer in the western blot experiment detecting MCM5 expression in HeLa and Jurkat whole cell lysates and it was further validated for immunofluorescence experiments in (Sedlackova et al., Nature, 2020 Oct 21; 587, 297-302).

MCM6 #1 (rabbit, Abcam, ab201683, clone EPR17686, 1:1,000) was previously validated by the manufacturer in the western blot experiment detecting MCM5 immunoprecipitated from HeLa cells and it was further validated for immunofluorescence experiments in (Lee et al., Nat Commun, 2021 May 5;12(1):2525).

MCM6 #2 (mouse, Novus Biologicals, H00004175-M04, clone 7D8, 1:1,000) was previously validated by the manufacturer in the western blot experiment detecting MCM6 in transfected and un-transfected lysates of 293T cell line and it was further validated for immunofluorescence experiments by QIBC in this study (Supplementary Fig. 2 and 4).

MCM7 #1 (mouse, Santa Cruz, sc-9966, clone 141.2, 1:1,000) was previously validated by the manufacturer in the western blot experiment detecting MCM7 expression in whole cell lysate samples of various cell lines and cited in more than 159 research papers (for immunofluorescence see e.g. Somyajit et al, Science 2017 Nov 10;358(6364):797-802.; Sedlackova et al., Nature, 2020 Oct 21; 587, 297-302).

PCNA (human, Immuno Concepts, 2037, 1:1,000) was validated for immunofluorescence experiments by QIBC in previous studies e.g. Somyajit et al, Science 2017 Nov 10;358(6364):797-802.; Spies et al, Nat Cell Biol. 2019 Apr;21(4):487-497, Zonderland et al, Mol Cell. 2022 Sep 15;82(18):3350-3365.e7.

POLD1 (rabbit, Abcam, ab186407, clone EPR15118, 1:1,000) was previously validated by the manufacturer in the western blot experiment detecting POLD1 expression in whole cell lysate samples of various cell lines and it was further validated in this study (Supplementary Fig. 1d) by western blot of naïve U2OS cells and U2OS cell line with GFP-tagged POLD1.

POLE1 (rabbit, Abcam, ab226848, 1:1,000) was previously validated by the manufacturer in the western blot experiment detecting POLE1 expression in whole cell lysate samples of the K562 cell line and it was further validated in this study (Supplementary Fig. 1e) by western blot of naïve U2OS cells and U2OS cell line with GFP-tagged POLE1.

TIMELESS (rabbit, Abcam, ab109512, clone EPR5275, 1:500) was previously validated in Somyajit et al, Science 2017 Nov 10;358(6364):797-802 in the western blot experiment detecting TIMELESS expression in whole cell lysates of U2OS cells treated with control siRNA or siRNA against TIMELESS and immunofluorescence experiments using QIBC.

Eukaryotic cell lines

Policy information about cell lines and Sex and Gender in Research

Cell line source(s)	Parental U2OS cell (ATCC HTB-96).<br>CDC45-mEGFP; POLD1-mEGFP; POLE1-mEGFP; CDC45-mEGFP, MCM4-HALO; MCM2-mEGFP are derivatives of the parental U2OS cell line.<br><br>Parental hTERT-RPE1 cell line (ATCC CRL--4000).<br>MCM4-Halo is derivative of the parental RPE-1 cell line.<br><br>CHO cell line ectopically expressing MCM4-mEmerald and PCNA-RFFP was kind gift from prof. David Gilbert (San Diego Biomedical Institute, USA). The origin and generation of this cell line is described in Kuipers et al, J Cell Biol. 2011 Jan 10;192(1):29-41.
Authentication	The parental U2OS and hTERT-RPE1 cell lines were authenticated by STR profiling. No further authentication of cell lines was performed.
Mycoplasma contamination	All cell lines are routinely (monthly basis) tested for mycoplasma (MycoAlert, Lonza) and always found negative.

Commonly misidentified lines (See ICLAC register)	No commonly misidentified cell lines were used in this study.
