# Supplementary material for: Solving the MCM paradox by visualizing the scaffold of CMG helicase at active replisomes
Source: Nat Commun. 2022 Oct 14;13:6090. doi: 10.1038/s41467-022-33887-5 (PMC9568601; doi:10.1038/s41467-022-33887-5)

Supplementary Figure 1a

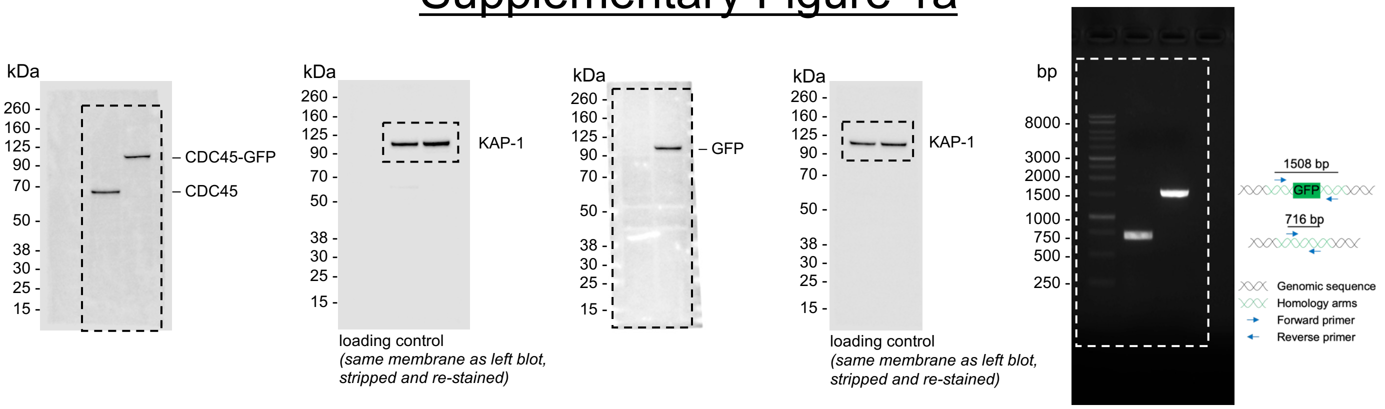

Supplementary Figure 1d

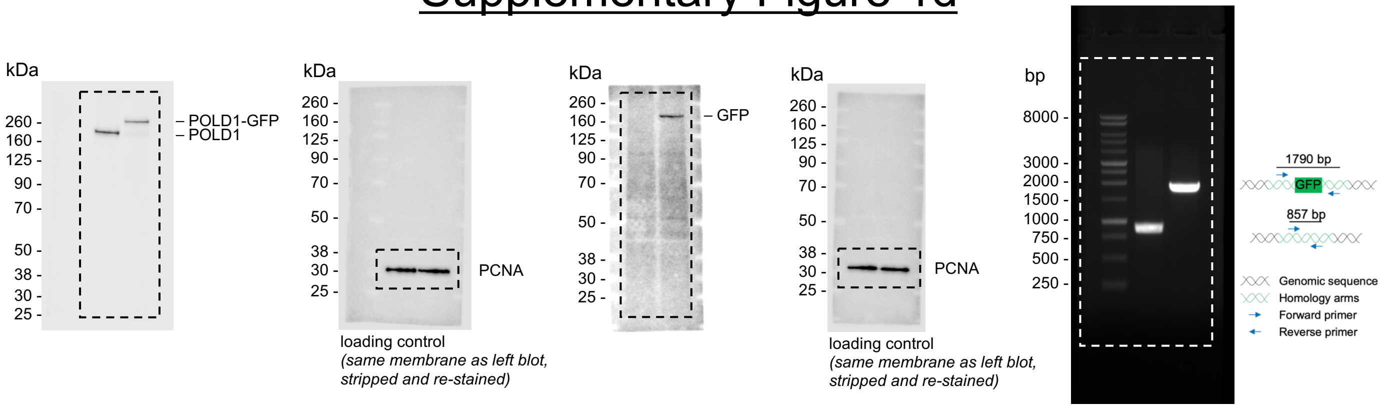

Supplementary Figure 1e

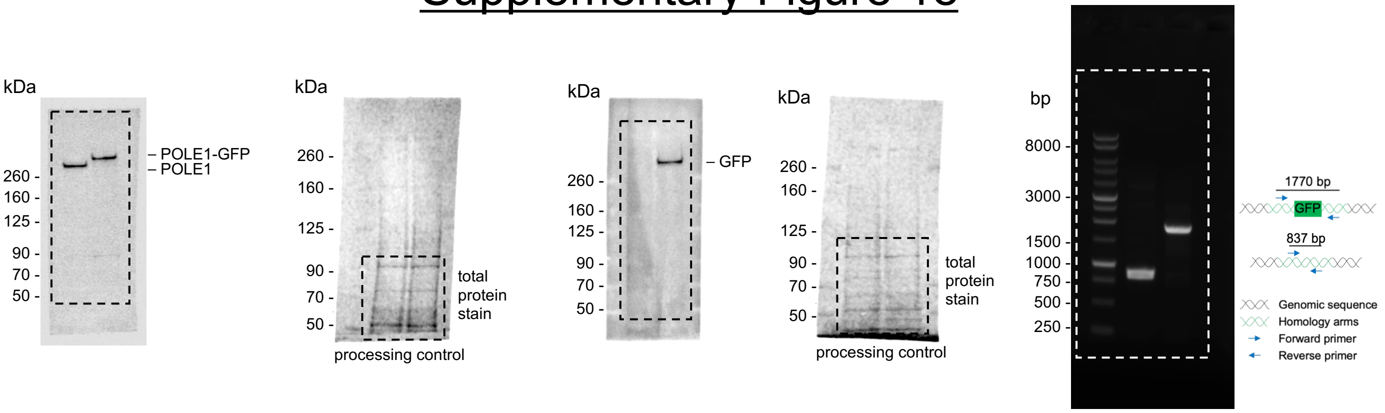

Supplementary Figure 7c

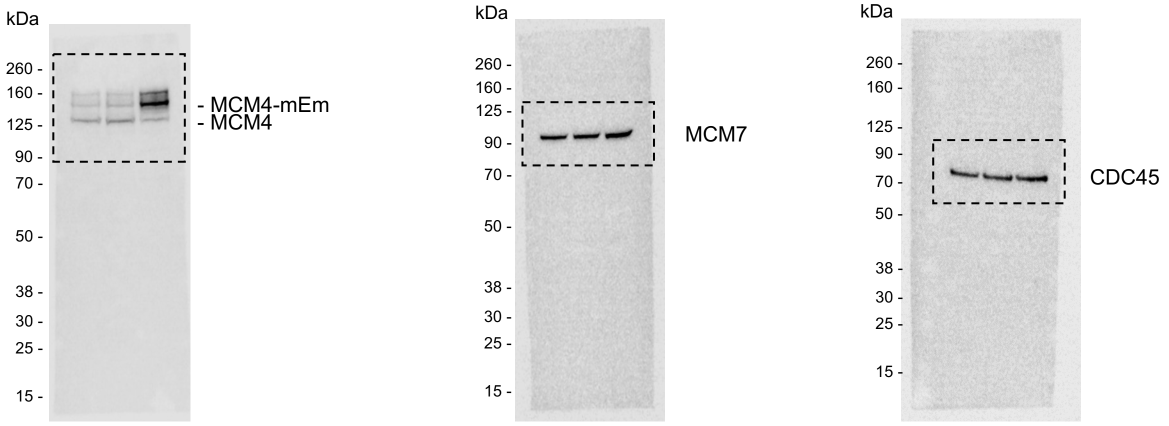

Supplementary Figure 7f

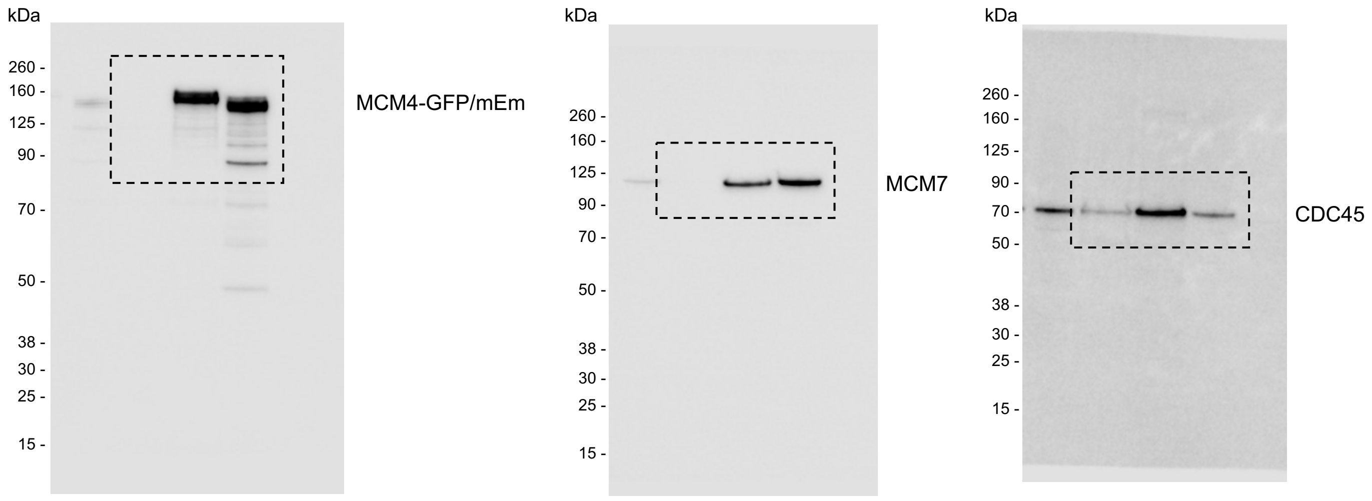

Supplementary Figure 9a

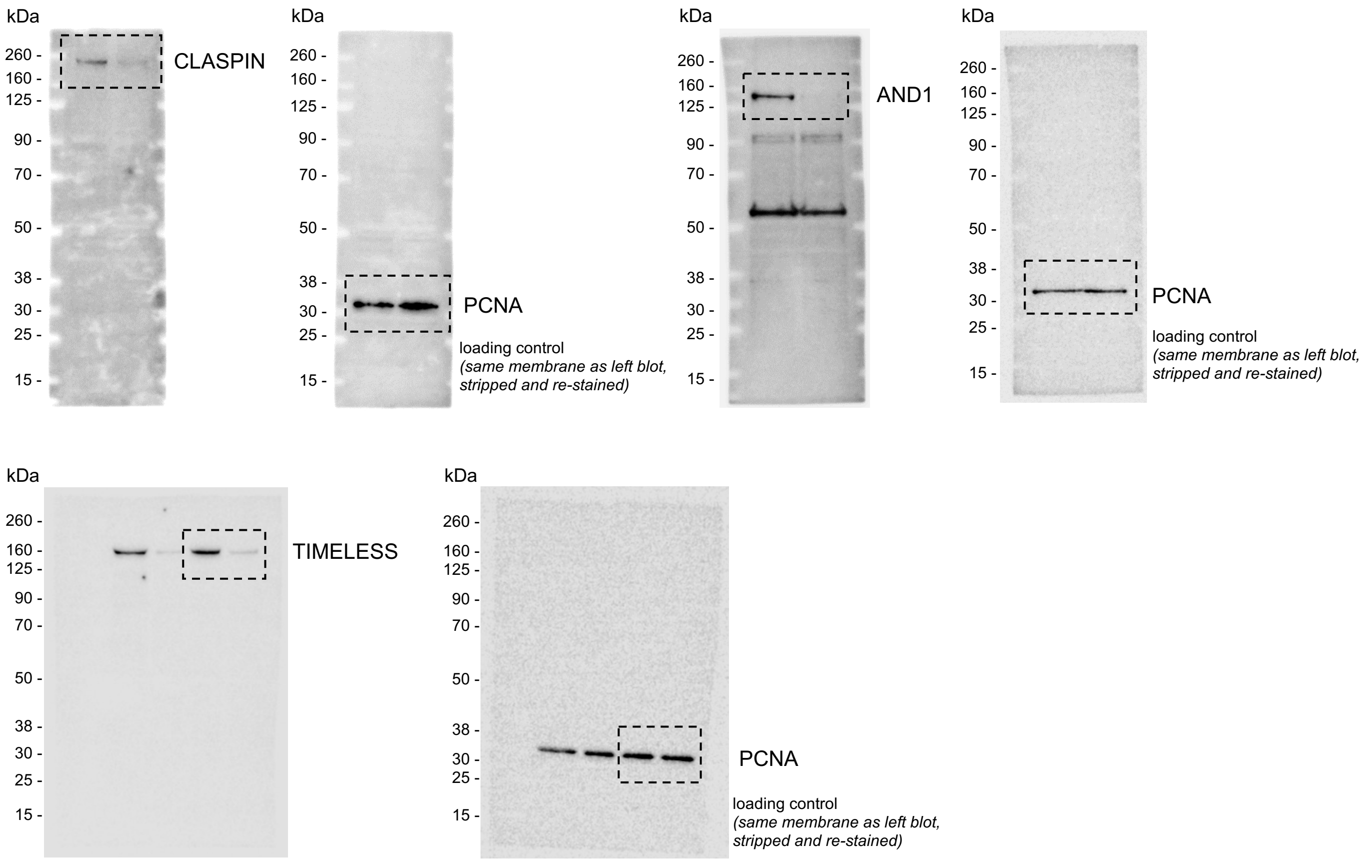

Supplement: Supplementary file 4 — Source Data [file 41467_2022_33887_MOESM4_ESM.zip › Polasek-Sedlackova et al_SOURCE DATA_final revision/Uncropped scans of all blots and gels.pdf]
